# Excluding pregnancy among women initiating antiretroviral therapy: efficacy of a family planning job aid

**DOI:** 10.1186/1471-2458-10-249

**Published:** 2010-05-14

**Authors:** Kwasi Torpey, Lona Mwenda, Mushota Kabaso, Thierry Malebe, Patrick Makelele, Francis Mwema, Henry Phiri, Jonathan Mukundu, Mark A Weaver, John Stanback

**Affiliations:** 1 Family Health International/Zambia Plot 2055 Nasser Road, Lusaka, Zambia

## Abstract

**Background:**

Guidelines for initiating ART recommend pregnancy testing, typically a urine test, as part of the basic laboratory package. The principal reason for this recommendation is that Efavirenz, a first-line antiretroviral medication, has the potential of causing birth defects when used in the first trimester of pregnancy and is therefore contraindicated for use by pregnant women. Unfortunately, in many African countries pregnancy tests are not routinely provided or available in ART clinics, and, when available outside clinics, are often not affordable for clients.

Recently, the World Health Organization added a family planning job aid called the 'pregnancy checklist,' developed by researchers at Family Health International, as a recommended tool for screening new ART clients to exclude pregnancy. Although the checklist has been validated for excluding pregnancy among family planning clients, there are no data on its efficacy among ART clients.

This study was conducted to assess the clinical performance of a job aid to exclude pregnancy among HIV positive women initiating ART.

**Methods:**

Non-menstruating women eligible for ART were enrolled from 20 sites in four provinces in Zambia. The pregnancy checklist was administered followed by a urine pregnancy test as a reference standard. Sensitivity, specificity, and positive and negative predictive values were estimated.

**Results:**

Of the 200 women for whom the checklist ruled out pregnancy, 198 were not pregnant, for an estimated negative predictive value of 99%. The sensitivity of the checklist was 90.0%, and specificity was 38.7%. Among the women, 416 out of 534 (77.9%) did not abstain from sex since their last menses. Only 72 out of the 534 women (13.4%) reported using reliable contraception. Among the 416 women who did not abstain, 376 (90.4%) did not use reliable contraception.

**Conclusion:**

The pregnancy checklist is effective for excluding pregnancy in many women initiating ART, but its moderate sensitivity and specificity precludes its use to completely replace pregnancy testing. Its use should be encouraged in low resource settings where pregnancy tests are unavailable or must be rationed. Family planning methods should be available and integrated into ART clinics.

## Background

Antiretroviral therapy (ART) has significantly improved the prognosis of individuals with HIV-1 disease in the developed world [1-3]. National Zambian ART guidelines and the World Health Organization (WHO) currently recommend two nucleosides and two non-nucleosides as first line regimens in the treatment of HIV patients. Nevirapine and Efavirenz are the non-nucleosides of choice [4,5]. More than 50% of patients on ART are women [6,7] and it is necessary that their needs are taken into consideration in the provision of care.

Guidelines for initiating ART recommend pregnancy testing, typically a urine test, as part of the basic laboratory package. The principal reason for this recommendation is that Efavirenz, a first-line antiretroviral medication, has the potential of causing birth defects when used in the first month of pregnancy and is therefore contraindicated for use by pregnant women [8]. Though pregnancy tests are supposed to be offered to all women of reproductive age initiating antiretroviral therapy, the reality in many African countries is that these tests are not routinely available in ART clinics, or their price puts them out of reach of many women. As a result, many women are currently offered treatment without a pregnancy test, thus compromising the quality of care offered to women accessing ART services. There is an urgent need for a tool to help exclude pregnancy in patient populations where pregnancy tests are often unavailable. Such a tool, if sufficiently accurate, could also be used to screen out women who are almost certainly not pregnant as a means to ration pregnancy test kits in situations where they are in short supply.

Recently, the WHO added a family planning job aid called the 'pregnancy checklist,' developed by researchers at Family Health International, as a recommended tool for screening new ART clients to exclude pregnancy [9]. The checklist has been validated for excluding pregnancy among family planning clients in Kenya, Nicaragua, and Egypt, and has been widely recommended as a tool to increase access to services [10-12]. In the validation study conducted in seven clinics in three regions of Kenya, the estimated negative predictive value was more than 99% and the estimated specificity was 89% [10]. In the Nicaragua and Egypt studies, the estimated negative predictive values were 100% and 99.6% respectively [11,12]. However there are no data to demonstrate the checklist's efficacy among ART clients.

The pregnancy checklist consists of six yes/no questions asked of non-menstruating women about last birth and menstrual period, duration and frequency of breastfeeding, recent abortion or miscarriage, abstinence from sexual relations, and current contraceptive use (Additional file 1). The questions are based on WHO-approved criteria [13] and are worded so that a positive response to any one is sufficient to rule out pregnancy, if the woman is free of signs or symptoms of pregnancy.

The primary objective of this study was to assess the clinical performance of the checklist as a screening tool for excluding pregnancy in women initiating antiretroviral therapy. A secondary objective was to assess the use of reliable contraception among this population.

## Methods

### Study Sites

Four ART sites each from Northern, Northwestern, Luapula, Central and Copperbelt Provinces were selected making a total of 20 sites. The convenience sample of sites in each province included urban and rural hospitals and health centers. Non-menstruating women eligible for ART in the 20 sites were consecutively enrolled from 1^st ^March 2009 to 30^th ^June 2009. The pregnancy checklist was administered to consenting eligible clients followed by a urine pregnancy test as a reference standard to assess validity.

### Eligibility criteria

All women initiating ART were considered for inclusion. The criteria for ART initiation were as follows: CD4 less than 200, WHO Stage IV regardless of CD4, or WHO Stage III and CD4 less than 350. Women who self-reported as pregnant, currently menstruating or who were in WHO Stage I, II, or III with no CD4 count were excluded from the study.

### Sample size calculation

It was determined that a minimum sample size of 426 would be required to have at least 80% power to reject the null hypothesis that the negative predictive value is lower than 98% at the one-sided 5% significance level. This calculation assumes that the true specificity for the checklist would be 89%, the value observed by Stanback et al. [10], that the true negative-predictive value would be at least 99.5% in this population, and that the pregnancy rate would be 2%.

### Data collection and entry

After obtaining informed consent, nurses at the ART clinic administered the checklist to eligible clients and then performed a urine dipstick pregnancy test. The data collection forms were reviewed for completeness and accuracy and then entered in a central Microsoft Access database. Client information collected included age, breastfeeding status, parity, CD4 count and menstrual status. The clients' yes/no responses to the six standard questions on the checklist were also collected.

### Statistical measures, definitions, relevance and analysis

For the purposes of this study, we define sensitivity to be the proportion of reference test (pregnancy test) positives for whom the pregnancy checklist would not rule out pregnancy. We define specificity to be the proportion of reference test negatives for which the checklist would rule out pregnancy. We further define the negative predictive value to be the proportion of patients who have a negative pregnancy test among those for whom the checklist would rule out pregnancy, and we define the positive predictive value to be the proportion of patients with a positive pregnancy test among those for whom the checklist would not rule out pregnancy.

The clinically relevant statistic is the negative predictive value since that statistic reflects the confidence the provider can have in the checklist's ability to rule out pregnancy for a given patient.

A cross-classification of patients with respect to pregnancy status and checklist result to estimate sensitivity, specificity, positive and negative predictive values was used along with exact 90% confidence intervals (two-sided 90% confidence intervals were used in order to obtain one-sided 95% lower confidence bounds, which are equivalent to one-sided tests at the 5% level).

### Ethical approval

Ethical Approval was granted by the ERES Converge Ethical Review Board in Lusaka, Zambia and the Protection of Human Subjects Committee of Family Health International, North Carolina, U.S.A with final clearance from the Ministry of Health in Zambia.

## Results

535 women initiating ART were enrolled into the study. One was subsequently excluded due to absence of pregnancy test results.

### Patient characteristics

The mean age of the women was 30.1 yrs with a mean CD4 count of 148.5 cells/ml. About 55% of the women were in WHO Stage III, with WHO Stages I and II accounting for approximately 14% and 19%, respectively. Twenty-three percent of women had non-postpartum amenorrhoea most likely due to HIV illness compared to only 7% with postpartum amenorrhoea. Only 13% of the patients did not have any children.

### Excluding pregnancy

Twenty-two women (4%) were found to be pregnant, for 20 of whom the checklist correctly did not rule out pregnancy. Of 200 women for whom pregnancy *was *ruled out by the checklist, 198 were in fact not pregnant. (Table 1)

**Table 1:** Comparing checklist results to dipstick pregnancy test.

Checklist	Dipstick Test		Total
	**Pregnant**	**Not Pregnant**	

Pregnancy not ruled out	20	314	334

Pregnancy ruled out	2	198	200

Total	22	512	534

Negative predictive value: 198/200 × 100 = 99.0% Positive predictive value:20/334 × 100 = 6.0%

Sensitivity:20/22 × 100 = 90.9% Specificity:198/512 × 100 = 38.7%

Taking the dipstick pregnancy result as the standard, the estimated sensitivity of the checklist was 90.9% (exact 90% CI: 74.1%, 98.4%), specificity 38.7% (35.1%, 42.4%), positive predictive value 6.0% (4.0%, 8.6%), and negative predictive value 99.0% (96.9%, 99.8%). Based on the estimated lower confidence bound, we can be 95% confident that the true negative predictive value would be at least 97% in similar populations, but we are unable to reject the null hypothesis specified for sample size calculations.

### Abstinence and use of reliable contraception

About 77.9% (416) of the women did not abstain from sex since their most recent menses. Only 72 out of the 534 women (13.4%) reported using reliable contraception. Among the 416 women who did not abstain, 376 (90.4%) did not use reliable contraception. A majority of the sexually active women who were not using reliable contraception were in WHO III (57.9%) or had CD4 less than 200 (77.6%)

## Discussion

Excluding pregnancy among women of reproductive age who present for ART is a challenge, especially in rural areas where pregnancy test kits may not be easily available or affordable. In this population of women eligible for ART, the negative predictive value of the checklist, i.e., its ability to accurately clinically rule out pregnancy was 99%. That is, as a clinical screening tool, the checklist was able to accurately distinguish those women who were almost certainly not pregnant from those for whom uncertainty remained; this latter group should be offered a pregnancy test before being provided with Efavirenz, but, in the event that pregnancy tests are unavailable, Nevirapine would be the safer option. Our estimated negative predictive value is similar to the estimates from earlier studies done in family planning clinics in Kenya, Nicaragua and Egypt [10-12]. Thus, providers can be confident when providing ARVs to a woman with a 'Not Pregnant' result from the checklist. However, fewer women in this sample of ARV patients met any of the six criteria that preclude possible pregnancy than in the Kenya and Egypt studies involving family planning clients [10,12]. This is likely due to the fact that many new family planning clients present for services during the extended postpartum period, and are thus more likely than ARV patients to be protected from pregnancy either by lactational infecundability or by postpartum sexual abstinence. As a result, the checklist was only able to exclude pregnancy in 39% of the ARV clients, but even a 39% reduction is not insignificant from a resource allocation perspective.

When pregnancy testing presents economic or logistic difficulties, the pregnancy checklist is a useful tool to enable correct provision of first line anti-retroviral drugs for many women. Where pregnancy cannot be ruled out after the use of the checklist, the health care worker must either refer the woman for a pregnancy test or avoid regimens containing Efavirenz. This checklist will help clinicians make sound clinical decisions and avoid potential teratogenicity even in the face of severely constrained resources.

The recent WHO Guidelines recommends initiation of ART when CD4 is less than 350 cell/ml [4]. CD4 is however required for stage I and II patients before therapy can be initiated. The previous guidelines recommended initiation of therapy in stage IV patients regardless of CD4, Stage III with CD4 less than 350 and Stage I and II with CD4 less than 200 [8]. The recent recommendation will expand the pool of patients with CD4 between 200 and 350. However, this is not expected to change the efficacy the checklist to rule out pregnancy considering the findings from the family planning clinics in Kenya, Nicaragua and Egypt where clients were expected to have higher CD4. With the reported toxicity in the use of Nevirapine in women with CD4 more than 250 [14,15] there is likely to be an increase in the use of Efavirenz. Tools to exclude pregnancy before Efavirenz use will be invaluable. In addition, the use of the checklist as a screening tool to exclude pregnancy in women who are already on Efavirenz and come to the clinic follow up visits, particularly as they become healthier and more sexually active may be very useful.

One unexpected finding was of the women interviewed; only 13% reported using reliable contraception. Of those who were sexually active, 90% did not use any reliable contraception. This finding is important because prevention of unwanted pregnancies among HIV positive patients is a major prong in the strategies to prevention of mother to child transmission. Unfortunately family planning needs for HIV positive patients are often overlooked in care and treatment programming.

One study of Rwandan women testing positive for HIV showed a 30% pregnancy rate. Seventy-four percent of these pregnancies were unintended, and most of the women surveyed were not using modern contraception [16,17]. There is evidence that use of ART may contribute to increased fertility and fertility desires among HIV positive women [18-21]. This calls for aggressive and systematic integration of family planning services in HIV prevention, care and treatment services.

There are some limitations to this study. It only enrolled women who met the MOH program's criteria for ART eligibility. However, the use of the pregnancy checklist in asymptomatic HIV positive women may not necessarily have the same negative predictive value. Another limitation is our use of dipstick pregnancy tests as the reference or "gold" standard. Such tests are highly accurate within a week after a missed period, but may not detect early pregnancies. Finally, because our results were based on a convenience sample, they may not strictly generalize to any larger population of women.

## Conclusion

The pregnancy checklist is effective in excluding pregnancy for many women, and a necessary adjunct to services when tests are scarce and/or expensive to help clinicians prescribe safe first line regimens to women. However, its moderate sensitivity and specificity preclude its use to completely replace pregnancy testing. It is important to integrate and promote use of reliable contraception among HIV patients.

## Competing interests

The authors declare that they have no competing interests.

## Authors' contributions

KT, JS conceived the study, participated in the design and helped draft the manuscript. LM, MK, TM, PM, FM, HP, JM and MAW participated in the design and helped draft the manuscript. MAW, JM and MK did the statistical analysis. All authors read and approved the final manuscript.

## Pre-publication history

The pre-publication history for this paper can be accessed here:

http://www.biomedcentral.com/1471-2458/10/249/prepub

## Supplementary Material

Additional file 1 **Checklist to rule out pregnancy**. Six questions in the checklist to exclude pregnancy Click here for file

## References

[B1] Brodt HR Kamps BS Gute P Knupp B Staszewski S Helm EB Changing incidence of AIDS defining illnesses in the era of antiretroviral combination therapy AIDS 1997 14 1731 1738 10.1097/00002030-199714000-00010 9386808

[B2] Egger M Hirschel B Francoli P Sudre P Wirz M Flepp M Rickenbach M Malinverni R Vernazza P Battegay M Impact of new antiretroviral combination therapies in HIV infected patients in Switzerland: Prospective Multicentre Study Swiss Cohort Br Med J 1997 315 1194 99 10.1136/bmj.315.7117.1194 PMC2127760 9393221

[B3] Egger M May M Chene G Phillips AN Ledergerber B Dabis F Costagliola D D'Arminio M de Wolf F Reiss P Lundgren JD Justice AC Staszewski S Leport C Hogg RS Sabin CA Gill MJ Salzberger B Sterne JA Prognosis of HIV infected patients starting highly active antiretroviral therapy: a collaborative analysis of prospective studies Lancet 2002 360 119 129 10.1016/S0140-6736(02)09411-4 12126821

[B4] World Health Organization Rapid Advice: Antiretroviral therapy for adults and adolescents 2009 WHO, Geneva

[B5] Ministry of Health/Zambia Antiretroviral Therapy for Chronic HIV infection in adults and adolescents 2007 Lusaka, Zambia

[B6] World Health Organization Towards Universal Access: Progress Report 2009 WHO, Geneva

[B7] Zambia Prevention Care and Treatment Partnership End of Program Report 2004-2009 to USAID 2009 Lusaka, Zambia

[B8] WHO Antiretroviral Therapy for Adults and Adolescents in Resourced Limited Settings: Towards Universal Access. Recommendations for Public Health Approach 2006 Revisions 2006 Geneva, Switzerland. World Health Organization

[B9] World Health Organization Chronic HIV care with ART and prevention: Integrated Management of Adolescents and Adult Illness, Integrated Management of Childhood Illness interim guidelines for health workers at health centre or district hospital outpatient clinic 2007 WHO, Geneva

[B10] Stanback J Qureshi Z Sekadde-Kingondu C Gonzalez B Nutley T Checklist For Ruling Out Pregnancy Among Family Planning Clients in Primary Care Lancet 1999 354 9178 566 10.1016/S0140-6736(99)01578-0 10470704

[B11] Stanback J Nanda K Ramirez Y Rountree W Validation of a Job Aid to Rule Out Pregnancy among Family Planning Clients in Nicaragua Pan Am Journal of Public Health 2008 23 2 116 8 10.1590/s1020-49892008000200007 18371282

[B12] Hassan EO Stanback J Eldamanhoury H "Validation of the Pregnancy Checklist in Selected Egyptian Family Planning Centers," Final Report 2005 Egyptian Fertility Care Foundation

[B13] World Health Organization Selected Practice Recommendation for Contraceptive use 2004 WHO, Geneva

[B14] Sanne I Mommeja-Marin H Hinkle J Bartlett JA Lederman MM Maartens G Wakeford C Shaw A Quinn J Gish RG Rousseau F Severe Hepatoxicity associated with Nevirapine Use In HIV infected subjects J Infect Dis 2005 191 825 829 10.1086/428093 15717255

[B15] Food and Drug Administration (FDA) Public Health Advisory for Nevirapine 2005 US FDA

[B16] Bangendaye L Pregnancy, Fertility desires, and contraceptive use among HIV infected women: findings from a survey of women enrolled in PMTCT HIV implementers meeting June 2008 Kampala, Uganda Abstract # 630

[B17] Bangendaye L Pregnancy, Fertility desires, and contraceptive use among HIV infected women enrolled in PMTCT in Rwanda: Final Report 2009 Family Health International/Rwanda

[B18] Myer L Carter R Toro P El-Sadr W Abrams E Impact of Antiretroviral Therapy on Pregnancy Among HIV Infected Women in sub-saharan Africa: A Multi-country analysis from MTCT Plus Initiative17th International AIDS Conference, Mexico City, Mexico. 3-8 August 2008 Abstract No. LBPE 1158

[B19] Homsy J Bunnel R Moore D King R Malamba S Nakityo R Glidden D Tappero J Mermin J Reproductive Intentions and Outcomes among women on Antiretroviral Therapy in Rural Uganda: A prospective cohort study PLOS One 4 1 e4149 10.1371/journal.pone.0004149 19129911 PMC2612743

[B20] Allen S Serufilira A Gruber V Kegeles S Perre P Van de Carael M Coates TJ Pregnancy and contraception use among urban Rwanda women after HIV testing and counseling Am J Public Health 1993 83 705 710 10.2105/AJPH.83.5.705 8484453 PMC1694707

[B21] Myer L Carter R Katyal M Toro P El-Sadr W Abrams E Impact of Antiretroviral Therapy on Pregnancy Among HIV Infected Women in sub-saharan Africa: A cohort study PLOS Med 2010 7 2 e1000229 10.1371/journal.pmed.1000229 20161723 PMC2817715

