# Supplementary material for: Excluding pregnancy among women initiating antiretroviral therapy: efficacy of a family planning job aid
Source: BMC Public Health. 2010 May 14;10:249. doi: 10.1186/1471-2458-10-249 (PMC2876108; doi:10.1186/1471-2458-10-249)
Supplement: Additional file 1 — Checklist to rule out pregnancy. Six questions in the checklist to exclude pregnancy [file 1471-2458-10-249-S1.DOC]

**Checklist to Rule Out Pregnancy (for clients without other contraindications)**

1. Have you given birth in the last four weeks?  No  Yes

2. Are you less than 6 months postpartum **and** fully breastfeeding **and** free

from menstrual bleeding since you had your child?  No  Yes

3. Did your last menstrual period start within the past seven days?  No  Yes

4. Have you had a miscarriage or abortion in the past seven days?  No  Yes

5. Have you abstained from sexual intercourse since your last menses?  No  Yes

6. Have you been using a reliable contraceptive method consistently and

correctly?  No  Yes

If the client answered **NO** to all of the questions, pregnancy cannot be ruled out. Client should await menses or use pregnancy test.

If the client answers YES to any of the questions and is free from signs and symptoms of pregnancy please provide her with the desired method
